# The prognostic significance of histologic variant on survival outcomes in patients with metastatic urothelial carcinoma receiving immune checkpoint inhibitor therapy

**DOI:** 10.1186/s12885-023-11398-w

**Published:** 2023-09-15

**Authors:** Tsung-Han Tsai, Po-Jung Su, Shih-Yu Huang, Ming-Chun Kuo, Chang-Ting Lin, Chia-Che Wu, Hao-Lun Luo, Chien-Hsu Chen, Chih-Chi Chou, Ting-Ting Liu, Chun-Chieh Huang, Kai-Lung Tsai, Yu-Li Su

**Affiliations:** 1 Division of Hematology Oncology, Department of Internal Medicine, College of Medicine, Kaohsiung Chang Gung Memorial Hospital, Chang Gung University, No.123, Dapi Rd., Niaosong Dist, Kaohsiung City, 833 Taiwan; 2 Division of Hematology Oncology, Chang Gung Memorial Hospital at Linkou and College of Medicine, Chang Gung University, Tao-Yuan, Kaohsiung City, Taiwan; 3 Department of Urology, College of Medicine, Kaohsiung Chang Gung Memorial Hospital, Chang Gung University, Kaohsiung City, Taiwan; 4 Department of Pathology, College of Medicine, Kaohsiung Chang Gung Memorial Hospital and Chang Gung University, Kaohsiung City, Taiwan; 5 Department of Radiation Oncology, College of Medicine, Kaohsiung Chang Gung Memorial Hospital, Chang Gung University, Kaohsiung City, Taiwan; 6 Department of Colorectal Surgery, College of Medicine, Kaohsiung Chang Gung Memorial Hospital, Chang Gung University, Kaohsiung City, Taiwan; 7 Genomic & Proteomic Core Laboratory, Department of Medical Research, Kaohsiung, Taiwan

**Keywords:** Metastatic urothelial carcinoma, Variant histology, Immune checkpoint inhibitors, Real-world data

## Abstract

**Background:**

While the treatment guidelines have been established for pure urothelial carcinoma (pUC), patients with variant type urothelial carcinoma (vUC) face limited effective treatment options. The effectiveness of immune checkpoint inhibitors (ICI) in patients with vUC remains uncertain and necessitates additional research.

**Method:**

We conducted a retrospective, multicenter study to explore the effectiveness of ICI in patients with pUC or vUC in Taiwan. We evaluated the overall response rate (ORR) through univariate logistic regression analysis and examined the overall survival (OS) and progression-free survival (PFS) using Kaplan-Meier analysis. Additionally, we employed univariate and multivariate Cox proportional hazards models to analyze the data.

**Result:**

A total of 142 patients (116 pUC, 26 vUC) were included in our final analysis. The ORR was marginally higher in patients with pUC compared to those with vUC (34.5% vs. 23.1%, p = 0.26). Among all patients, 12.9% with pUC achieved a complete response (CR) after ICI treatment, while no vUC cases achieved CR (p = 0.05). There were no significant differences in PFS (median 3.6 months vs. 4.1 months, p = 0.34) or OS (median 16.3 months vs. 11.0 months, p = 0.24) when comparing patients with pUC or vUC. In the subgroup analysis, patients with pUC who underwent first-line ICI treatment exhibited significantly improved OS compared to those with vUC (24.6 months vs. 9.1 months, p = 0.004).

**Conclusion:**

The use of ICI as monotherapy is a feasible and effective treatment approach for patients with metastatic vUC.

**Supplementary Information:**

The online version contains supplementary material available at 10.1186/s12885-023-11398-w.

## Introduction

Bladder cancer (BC) is a prevalent malignancy worldwide, with an estimated 573,000 new cases and 21,300 deaths reported annually in United State [1]. At the time of diagnosis, 25% of patients have muscle-invasive BC (MIBC), while 5% have metastatic disease [2]. Metastatic urothelial carcinoma (mUC) is a challenging disease due to aggressive behavior and high mortality rate. Currently, the standard first-line treatment for patients with mUC is cisplatin-based chemotherapy, including gemcitabine with cisplatin or dose-dense MVAC (methotrexate, vinblastine, doxorubicin and cisplatin). However, there are patients who are not suitable for cisplatin-based chemotherapy due to various reasons such as chronic kidney disease, poor performance status or congestive heart failure, and are therefore considered cisplatin-ineligible [3]. In such cases, carboplatin-containing chemotherapy may be a suitable alternative first-line treatment. Despite these aggressive treatments, the median overall survival (OS) for mUC ranges from 13 to 15 months with standard chemotherapy [4, 5]. The current treatment approach for mUC falls short of expectations, highlighting the ongoing necessity for the discovery of new drugs that offer improved effectiveness and tolerability.

The introduction of immune checkpoint inhibitor (ICI) therapy in 2017 has revolutionized the approach to treating mUC [6]. In particular, for patients who are unfit for platinum-based chemotherapy, ICI provides a ray of hope for treatment [7]. In the KEYNOTE 045 study, pembrolizumab demonstrated a superior survival benefit compared to chemotherapy in patients who were refractory to first-line platinum-based treatment [8]. Although the first-line use of ICI in mUC patients did not demonstrate superior survival to platinum-based chemotherapy in KEYNOTE 361 and IMVigor 130 studies, pembrolizumab has still been granted FDA approval for first-line use in patients who are ineligible for any platinum-based treatment [9, 10].

Over the past few years, the incidence of variant UC (vUC) has been increasing due to heightened awareness of its underlying pathology [9]. Earlier research has indicated that variant UC (vUC) exhibits a poorer postoperative recurrence-free survival (RFS), OS, and increased resistance to chemotherapy when compared to pure UC (pUC) [11–14]. In advance, limited and conflicting data exist regarding the effectiveness of ICI in treating vUC, making it challenging to establish a clear stance on the use of ICI in these rare cases [15–17]. Miller et al. conducted a study indicating that the overall response rate (ORR) and OS of ICI treatment were similar in both vUC and pUC cases [15]. Contrarily, the study conducted by Minato et al. demonstrated that vUC exhibited a higher ORR when treated with pembrolizumab [17]. Given the uncertain efficacy of ICI patients with vUC and the limited available data specifically for Asian population, we proposed a real-world study in Taiwan to investigate the treatment outcomes of ICI therapy in vUC patients.

## Material and method

### Patient selection and treatment

**Fig. 1:**
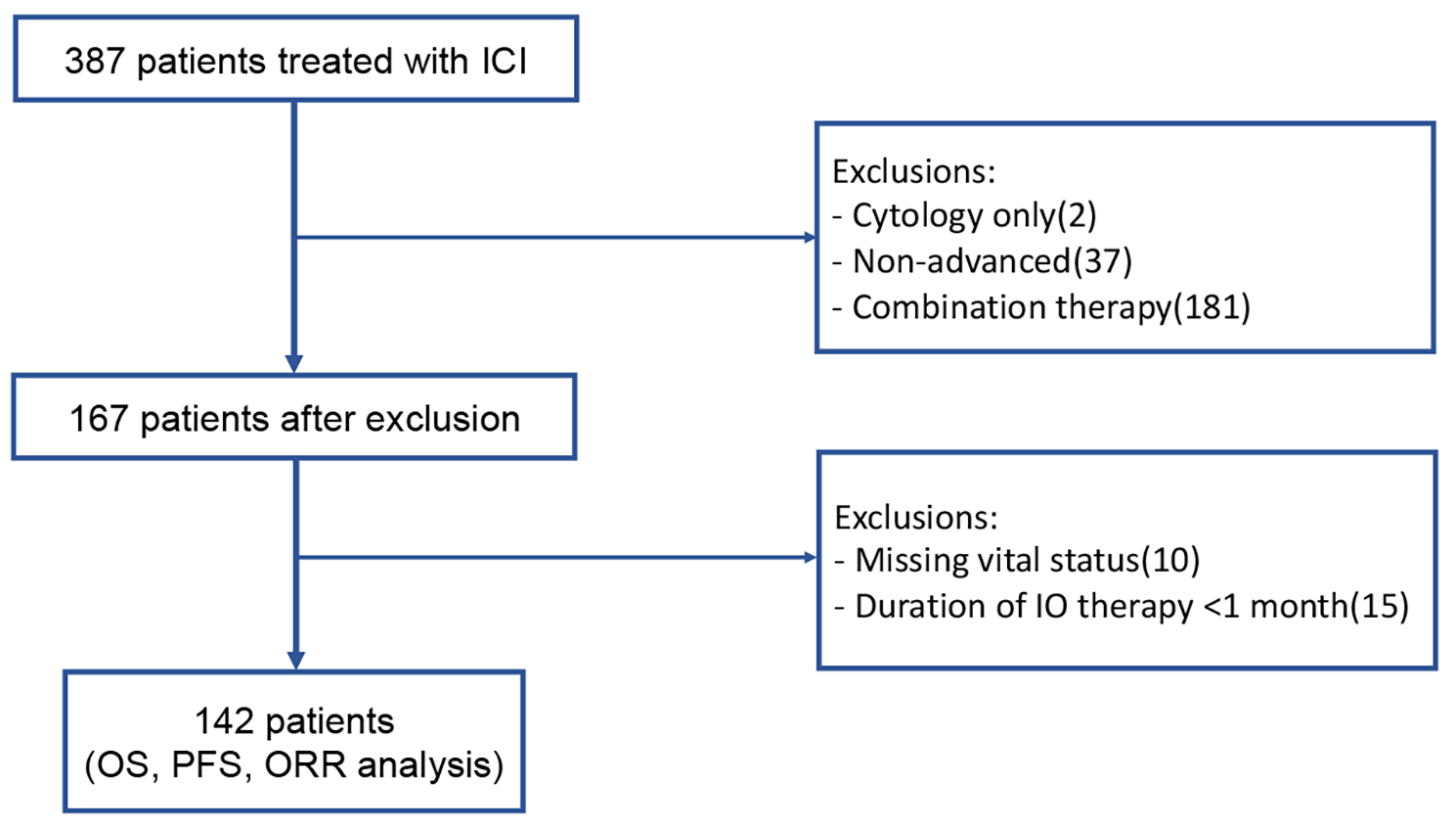
Consort diagram of the study

We conducted a retrospective, multicenter analysis from patients of two medical centers in Taiwan: Kaohsiung Chang Gung Memorial Hospital and Linkou Chang Gung Memorial Hospital. All patients had a definite histopathological diagnosis of urothelial carcinoma between August 2006 to April 2022. Every patient included in the study received a minimum of one cycle of ICI monotherapy, which consisted of pembrolizumab, nivolumab, avelumab, durvalumab, and atezolizumab. Patients with localized disease or ICI treatment duration less than 1 month were excluded from the study. It is essential to note that clinical signs of infection, such as fever, positive blood or urine cultures, and the absence of systemic inflammatory response syndrome (SIRS), are also required prior to ICI treatment. The detailed consort diagram can be found in Fig. 1.

### Clinical data and response evaluation

We extracted the following data from medical records, including age, sex, Eastern Cooperative Oncology Group (ECOG) performance status, laboratory results, Bajorin risk score, and PD-L1 expression level. The presence of metastasis in visceral organs was determined by computed tomography (CT) or magnetic resonance imaging (MRI). PD-L1 immunohistochemistry staining was performed using the Dako 22C3 anti-human PD-L1 antibody and interpreted by certified pathologists (T.T.L. and C.C.C.). Treatment response was assessed by clinicians following the RECIST guideline (version 1.1).

### Statistical design

To evaluate the heterogeneity among different histologic subgroups, a paired t-test or chi-square test was employed. We used Kaplan-Meier method with the log-rank test to estimate survival analysis (PFS and OS). Treatment subgroups were compared using hazard ratios (HRs) and associated 95% confidence intervals (CIs) based on an unstratified Cox regression model, considering both univariate and multivariate analysis. In all analyses, two-tailed tests were utilized, and statistical significance was determined as p-value < 0.05. The statistical analyses were performed using SPSS (version 26) and GraphPad Prism (version 9.5).

## Results

### Patient characteristics

A total of 387 mUC patients were included in the study. Patients with cytologic diagnosis only (2), non-advanced disease (37), received combination treatment with chemotherapy (181), had missing vital data (10) or had a treatment duration of less than 1 month (15) were excluded (Fig. 1). The final analysis included 142 eligible patients, consisting of 116 patients with pUC (82%) and 26 patients with vUC (18%).

The baseline demographic characteristics of the enrolled patients were well balanced across the different pathologic groups, as depicted in Table 1. The majority of patients were aged over 65 (66.9%), had an ECOG performance status of 0–1 (85.9%), and presented with lymph node metastasis (86.6%). A majority of the patients (54.2%) in the study had tumors originating from the upper urinary tract, while a significant proportion (52.8%) exhibited visceral metastasis. The utilization of anti-PD1 treatment was significantly higher in vUC patients compared to the pUC group (76.9% vs. 53.4%, p = 0.047).

**Table 1:** Basic characteristics of all patients

	All (*n*, %)	pUC (*n*, %)	vUC (*n*, %)	*P* value	
Age (year)				0.46	
< 65	47 (33.1)	40 (34.5)	7 (26.9)		
≥ 65	95 (66.9)	76 (65.5)	19 (73.1)		
Gender				0.72	
Female	59 (41.5)	49 (42.2)	10 (38.5)		
Male	83 (58.5)	67 (57.8)	16 (61.5)		
ECOG				0.83	
0–1	122 (85.9)	100 (86.2)	22(84.6)		
≥ 2	20 (14.1)	16 (13.8)	4 (15.4)		
Renal function (mL/min)				0.57	
CCr ≥ 60	46 (34.1)	37 (33)	9 (39.1)		
CCr < 60	89 (65.9)	75 (67)	14 (60.9)		
Primary site				0.93	
Upper tract	77 (54.2)	64 (55.2)	13 (50.0)		
Bladder	63 (44.4)	52 (44.8)	11 (42.3)		
Multifocal	2 (1.4)	0 (0)	2 (7.7)		
Lymph node metastasis				0.35	
No	19 (13.4)	17 (14.7)	2 (7.7)		
Yes	123 (86.6)	99 (85.3)	24 (92.3)		
Visceral metastasis				0.75	
No	67 (47.2)	54 (46.6)	13 (50.0)		
Yes	75 (52.8)	62 (53.4)	13 (50.0)		
Liver metastasis				0.66	
No	110 (77.5)	89 (76.7)	21 (80.8)		
Yes	32 (22.5)	27 (23.3)	5 (19.2)		
Lung metastasis				0.63	
No	87 (61.3)	70 (65.4)	17 (60.3)		
Yes	55 (38.7)	46 (34.6)	9 (39.7)		
Bone metastasis				0.25	
No	114 (80.3)	91 (78.4)	23 (88.5)		
Yes	28 (19.7)	25 (21.6)	3 (11.5)		
WBC (× 10^3^/µL)				0.36	
< 10	101 (71.1)	85 (73.3)	16 (61.5)		
≥ 10	32 (22.5)	25 (21.6)	7 (26.9)		
missing	9 (6.30)	6 (5.20)	3 (11.5)		
NLR				0.30	
< 3	45 (31.7)	36 (31.0)	9 (34.6)		
≥ 3	85 (59.9)	72 (62.1)	13 (50.0)		
missing	12 (8.5)	8 (6.9)	4 (15.4)		
Hemoglobin (g/dL)				0.35	
≥ 10	89 (62.7)	74 (63.8)	15 (57.7)		
< 10	45 (31.7)	37 (31.9)	8 (30.8)		
missing	8 (5.6)	5 (4.3)	3 (11.5)		
Bajorin prognostic factor				0.99	
0	58 (40.8)	47 (40.5)	20 (42.3)		
1	73 (51.4)	60 (51.7)	5 (50.0)		
2	11 (7.7)	9 (7.8)	1 (7.7)		
PD-L1 expression				0.27	
< 10	38 (26.8)	29 (25.0)	9 (34.6)		
≥ 10	40 (28.2)	31 (26.7)	9 (34.6)		
missing	64 (45.1)	56 (48.3)	8 (30.8)		
Line of therapy				0.29	
First-line	79 (55.6)	62 (53.4)	17 (65.4)		
≥ 2 line	63(44.4)	54 (44.6)	9 (34.6)		
ICI type				0.047	
Anti-PD1	82 (57.7)	62 (53.4)	20 (76.9)		
Anti-PDL1	60 (42.3)	54 (46.6)	6 (23.1)		
De novo metastasis				0.30	
No	80 (56.3)	63 (54.3)	17 (65.4)		
Yes	62 (43.7)	53 (45.7)	9 (34.6)		
Radical surgery^1^				0.09	
No	76 (53.5)	66 (56.9)	10 (38.5)		
Yes	66 (46.5)	50 (43.1)	16 (61.5)		
Systemic chemotherapy^2^				0.33	
Neoadjuvant	16 (24.2)	13 (26)	3 (18.8)		
Adjuvant	15 (22.8)	13 (26)	2 (12.5)		
None	35 (53)	24 (48)	11 (68.8)		

Abbreviations: pUC, pure urothelial carcinoma; vUC, variant urothelial carcinoma; CCr, clearance of creatinine; ECOG, Eastern Cooperative Oncology Group; NLR, neutrophil to lymphocyte ratio; WBC, white blood cell count; PD-L1, programmed cell death ligand-1; ICI, immune checkpoint inhibitor

^1^radical cystectomy or radical nephroureterectomy

^2^chemotherapy pre or post radical surgery

Among the variant types, squamous differentiation was the most prevalent (69.2%), followed by the micropapillary type (19.2%). A detailed breakdown of the distribution of histopathologic variants can be found in Table 2.

**Table 2:** The distribution of histopathologic variants among the patients

			All (*n*, %)	
Histology				
Squamous			18 (69.2)	
Micropapillary			5 (19.2)	
Sarcomatoid			1 (3.8)	
Adenocarcinoma^1^			1 (3.8)	
Small cell NEC			1 (3.8)	

NEC, neuroendocrine carcinoma

^1^Mucinous histology

### Treatment response and survival outcomes

When assessing the overall tumor response to ICI (shown in Table 3), it was observed that patients with pUC achieved a complete response rate of 12.9%, whereas no complete responses were observed among patients with vUC. Moreover, patients with pUC exhibited a higher ORR of 34.5% compared to 23.1% in patients with vUC. The disease control rate (DCR) was achieved in 54 patients with pUC (46.6%) and 12 patients with vUC (46.2%). These findings suggest differing response patterns between pUC and vUC in the context of ICI therapy.

**Table 3:** Efficacy of immune checkpoint inhibitor stratified by histologic variant

	pUC n (%)	vUC n (%)	*p* value
Complete response (CR)	15 (12.9)	0	0.05
Partial response (PR)	25 (21.6)	6 (23.1)	0.87
Stable disease (SD)	14 (12.1)	6 (23.1)	0.15
Progressive disease (PD)	62 (53.4)	14 (53.8)	0.96
Response rate (RR)	40 (34.5)	6 (23.1)	0.26
Disease control rate (DCR)	54 (46.6)	12 (46.2)	0.97

Abbreviations: pUC, pure urothelial carcinoma; vUC, variant urothelial carcinoma

**Fig. 2:**
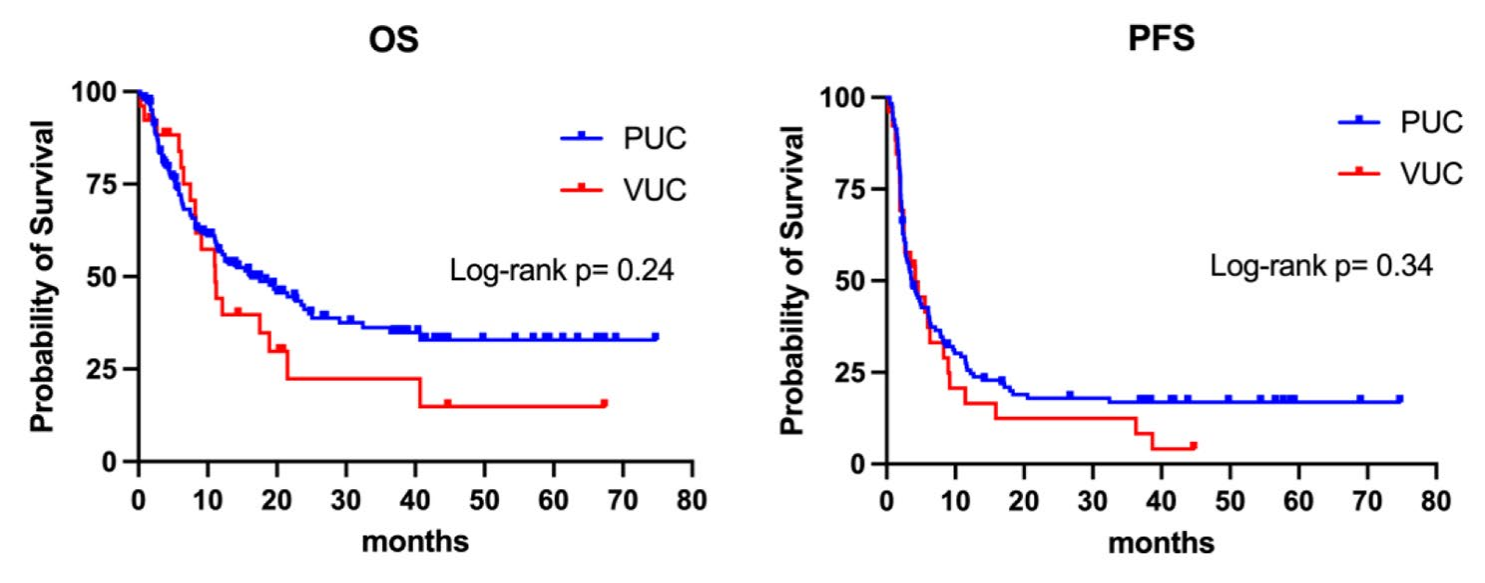
Kaplan-Meier survival curves of the pUC and vUC patients undergoing immune checkpoint inhibitor. Left: overall survival; Right, progression-free survival

During the median follow-up period of 37.2 months, a total of 79 deaths occurred (61 in the pUC group and 18 in the vUC group). The Kaplan-Meier curves for OS and PFS are depicted in Fig. 2. The median OS for patients with pUC and vUC was 16.3 months and 11.0 months, respectively (HR 0.74, 95% CI 0.42–1.30, p = 0.24). The median PFS was 3.6 months for pUC and 4.1 months for vUC (HR 0.80, 95% CI 0.50–1.30, p = 0.34). These findings suggest similar survival outcomes between pUC and vUC, although the observed differences did not reach statistical significance.

**Fig. 3:**
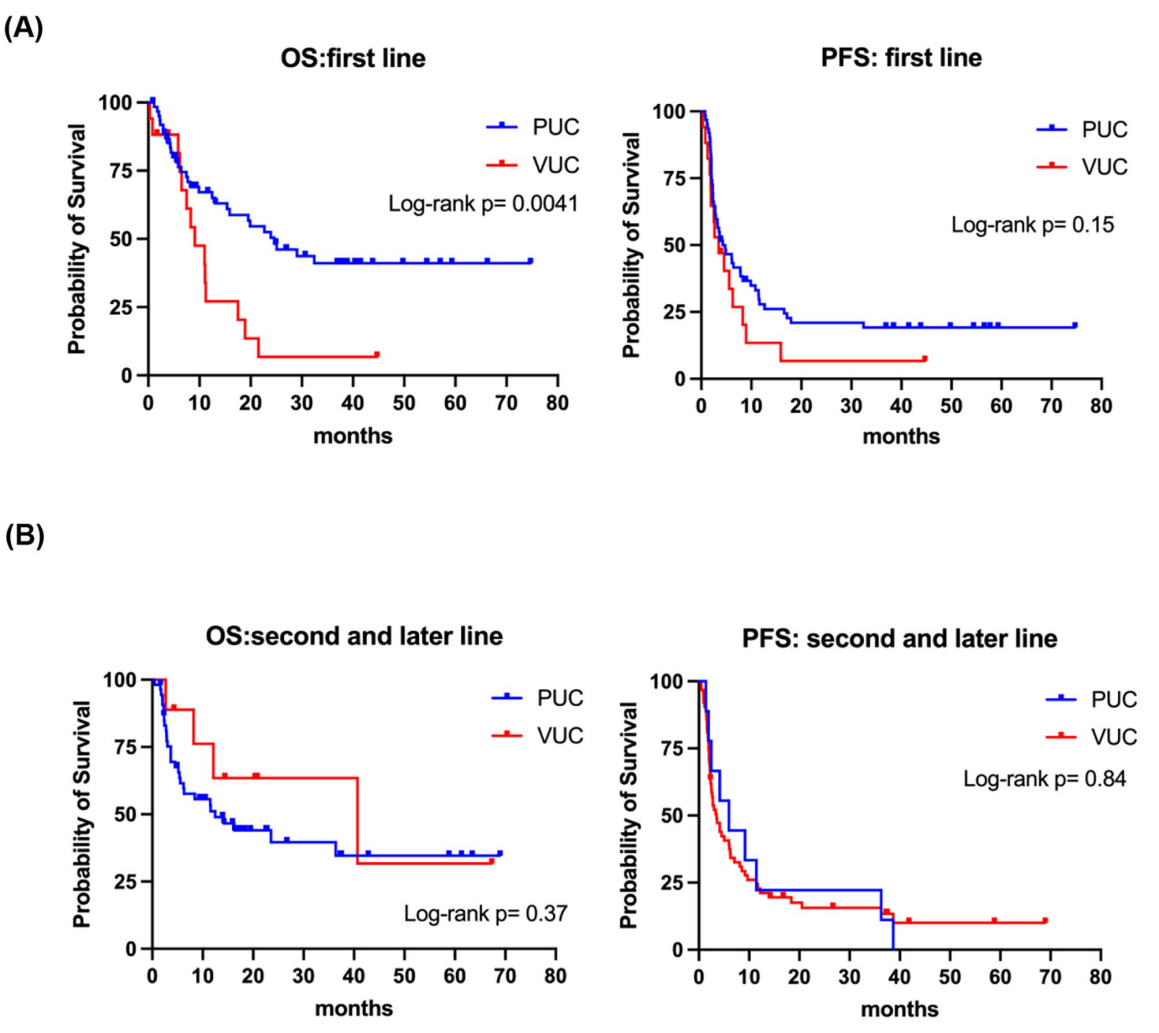
Kaplan-Meier survival curves of the pUC and vUC patients undergoing immune checkpoint inhibitor stratified by treatment sequence. (A) First-line ICI treatment (B) Second and later line ICI treatment

We further analyzed the survival outcomes based on different treatment lines (first-line vs. second and later line) and are presented in Fig. 3. In the first-line setting, patients with pUC exhibited a median OS of 24.6 months, while those with vUC had a median OS of 9.1 months (p = 0.004). The median PFS was 4.3 months for pUC and 3.4 months for vUC (p = 0.15). In the second and later line setting, pUC showed a median OS of 40.7 months, while vUC had a median OS of 12.4 months (p = 0.37). The median PFS was 3.5 months for pUC and 5.9 months for vUC (p = 0.84).

Tables 4 and 5 present the subgroup analysis for survival outcomes. In the univariate analysis, the significant prognostic factors for OS were Bajorin risk score (p < 0.001), baseline WBC (p < 0.001), and hemoglobin levels (p = 0.007). Multivariate analysis also revealed that the Bajorin risk score (p = 0.002), baseline WBC (p = 0.003), and hemoglobin levels (p = 0.003) were associated with improved overall survival. Regarding multivariate analysis of progression-free survival, the Bajorin risk score (p = 0.025) and baseline WBC (p = 0.01) were identified as significant prognostic factors.

**Table 4:** Univariate and multivariate analysis of PFS

Characteristics	Median PFS	Univariate			Multivariate	
	(month)	HR (95% CI)	*p* value		HR (95% CI)	*p* value
Age (year)			0.64			
< 65	4.1	1				
≥ 65	3.5	1.09 (0.74–1.60)				
Gender			0.42			
Female	4.1	1				
Male	3.6	1.16 (0.80–1.68)				
Bajorin risk score			0.007			0.025
0–1	4.3	1			1	
2	2.0	2.24 (1.22–4.09)			2.10(1.09–4.03)	
WBC (× 10^3^/µL)			< 0.001			0.001
< 10	5.9	1			1	
≥ 10	2.0	2.33 (1.52–3.59)			2.17(1.40–3.37)	
Hemoglobin (g/dL)			0.30			0.265
< 10	2.5	1			1	
≥ 10	4.5	0.80(0.53–1.21)			0.79(0.52–1.19)	
Origin			0.45			
Lower tract	6.0	1				
Upper tract	3.2	1.15(0.79 − 0.66)				
Histology			0.35			0.19
Non-variant	3.6	1			1	
Variant	4.1	1.23(0.79–1.93)			1.37(0.85–2.23)	
Line of therapy			0.51			
1	3.7	1				
≥ 2	3.5	1.13(0.78–1.63)				

Abbreviations: PFS, progression free survival; HR, hazard ratio; CI, confidence interval; ECOG, Eastern Cooperative Oncology Group; WBC, white blood cell count; NLR, neutrophil to lymphocyte ratio; Hb, hemoglobin

**Table 5:** Univariate and multivariate analysis of OS

Characteristics	Median OS	Univariate			Multivariate	
	(month)	HR (95% CI)	*p* value		HR (95% CI)	*p* value
Age (year)			0.80			
< 65	18.9	1				
≥ 65	15.9	0.94 (0.59–1.49)				
Gender			0.753			
Female	15.9	1				
Male	17.5	1.07 (0.68–1.68)				
Bajorin risk score			< 0.001			0.002
0–1	19.5	1			1	
2	3.3	3.16 (1.69–5.88)			2.94(1.49–5.79)	
WBC (× 10^3^/µL)			< 0.001			0.003
< 10	22.7	1			1	
≥ 10	6.5	2.38 (1.46–3.87)			2.14(1.29–3.56)	
Hemoglobin (g/dL)			0.007			0.003
< 10	7.4	1			1	
≥ 10	19.9	0.53(0.33–0.85)			0.47(0.29–0.78)	
Origin			0.36			
Lower tract	19.9	1				
Upper tract	12.4	1.23(0.78–1.94)				
Histology			0.16			0.182
Non-variant	19.9	1			1	
Variant	11.0	1.45(0.85–2.47)			1.48(0.83–2.64)	
Line of therapy			0.89			
1	17.5	1				
≥ 2	14.2	1.03(0.65–1.61)				

Abbreviations: PF, progression free survival; HR, hazard ratio; CI, confidence interval; ECOG, Eastern Cooperative Oncology Group; WBC, white blood cell count; NLR, neutrophil to lymphocyte ratio; Hb, hemoglobin

## Discussion

The impact of histologic variant on treatment response and survival outcomes in patients receiving ICI therapy remains a topic of ongoing debate. To address this, we conducted a retrospective study aiming to assess the effectiveness of ICI monotherapy in patients with pUC and vUC. Our findings revealed that the ORR was numerically higher in patients with pUC compared to those with vUC (34.5% vs. 23.1%). Notably, patients with pUC exhibited a more favorable depth of response, as evidenced by a higher complete response rate of 12.9%, while no complete responses were observed in vUC patients. Although our results showed similar PFS and OS between the two groups overall, subgroup analysis demonstrated that patients with pUC experienced improved OS in the context of first-line ICI treatment.

The findings of our study are consistent with several previous investigations. Miller et al. conducted a study that revealed similar ORR (28% vs. 29%, p = 0.9) and median OS (11 months vs. 10.1 months, p = 0.6) between patients with pUC and vUC who underwent ICI monotherapy [15]. In another study by Kobayashi et al., they found a comparable ORR between vUC and pUC patients (24.5% vs. 17.3%, p = 0.098), and no significant differences were observed in terms of PFS or OS between the two groups [16]. However, Minato et al. demonstrated higher ORR and complete response (CR) rates in vUC patients (59.1%, CR rate: 9.1%) compared to pUC patients (24.7%, CR rate: 4.9%) when treated with pembrolizumab monotherapy after platinum treatment failure, although there were no significant differences in PFS or OS [17]. A detailed comparison can be found on Table 6.

**Table 6:** Efficacy of ICI in vUC from published retrospective studies

Study	Patients	Treatment (line of therapy)	Gender	Origin	Liver metastasis	ORR (CR rate)	Median survival (months)
*Miller et al.*	120	Anti-PD-L1 and Anti-PD-1 (2nd or later line, mixed)	M: 71% F: 29%	Upper: 16% Lower: 84%	15%	29% (NA)	PFS: 5.2 OS: 10.1
*Kobayashi et al.*	147	Pembrolizumab (2nd line)	M: 75.2% F: 24.8%	Upper: 50.6% Lower: 49.4%	19.7%	24.5% (6.1%)	PFS: NA OS: 12.3
*Minato et al.*	22	Pembrolizumab (2nd line)	M: 81.8% F: 18.2%	Upper: 45.5% Bladder: 45.5% Multifocal: 9%	13.6%	59.1% (9.1%)	PFS: 10.4 OS: 23.8
*Our study*	26	Anti-PD-L1 and Anti-PD-1(mixed)	M: 61.5% F: 38.5%	Upper: 50% Bladder: 42.3% Multifocal: 7.7%	19.2%	23.1% (0%)	PFS: 4.1 OS: 11.0

M, male; F, female; ORR, overall response rate; NA, not available

Given the conflicting findings, our data contributes novel evidence to the appropriateness of employing ICI for the treatment of vUC. Of note, our research findings are the first to demonstrate that ICI yield significantly superior OS benefits in pUC compared to vUC in the first-line treatment of mUC. These results can provide guidance in the selection of appropriate patients for ICI treatment.

Different histologic variants can potentially influence the response to ICI in a clinical setting. The 2016 WHO classification identifies vUC as a histologic variant encompassing a diverse range of subtypes, such as squamous, glandular, micropapillary, sarcomatoid, plasmacytoid, small cell carcinoma, and others [18]. The squamous and micropapillary variants, among others, have been associated with more aggressive characteristics, resistance to chemotherapy, and poorer overall survival outcomes [19–22]. To gain insights into the response of specific variants to ICI, the most effective approach is to examine the ORR and pathologic response through neoadjuvant trials. The PURE-01 study observed that neoadjuvant pembrolizumab treatment in patients with muscle-invasive bladder cancer (MIBC) led to a substantial pathologic complete response (PCR) rate, with impressive outcomes of up to 42% [23]. Among all the enrolled variants, the squamous variant demonstrated tumor downstaging to pT1 or pTa in 86% of cases, with one case (14%) even achieving pCR [24]. The NABUCCO trial reported a 100% pCR rate in two out of two patients with the squamous variant who received neoadjuvant treatment with nivolumab and ipilimumab, suggesting that the squamous variant is not the determining factor for the effectiveness of ICI treatment [25]. Notably, the squamous variant exhibited a substantial presence of CD-274 gene amplification (5%), PD-L1 expression, and a high tumor mutational burden (TMB), all of which suggest its potential responsiveness to ICI treatment [26].

The lymphoepithelioma-like carcinoma (LELC) variant is a histologic subtype that closely resembles nasopharyngeal carcinoma and shares a connection with Epstein-Barr virus (EBV) activity [27]. A case series involving pulmonary LELC demonstrated a notable high response rate (80%) and a longer median PFS compared to standard chemotherapy when treated with ICI [28]. In the PURE-01 study, two out of three cases with LEL variants exhibited a pT0 response by neoadjuvant pembrolizumab treatment [24]. Urothelial carcinoma with sarcomatoid differentiation is a rare subtype characterized by advanced stage and associated with a worse survival [29]. Several case reports demonstrated that sarcomatoid variant had an exceptional response to ICI [30, 31]. In a study by Kobayashi et al., it was demonstrated that the sarcomatoid variant, when treated with ICI, exhibited a significantly higher ORR and improved OS compared to pUC [16]. The presence of sarcomatoid transformation in conventional bladder cancer is thought to entail a distinct mutational landscape and elevated PD-L1 expression. These findings provide supportive evidence for the potential effectiveness of ICI treatment [32].

When considering the depth of response to treatment, it should be noted that none of the vUC patients in our study achieved a clinical complete response (CR). However, other studies have reported clinical CR rates ranging from 6.1 to 9.1% [16, 17]. It is important to consider that the heterogeneity in patient backgrounds, various ICI agents used, and the small number of cases in these studies may be confounding factors. Nonetheless, our study found comparable disease control rates (DCR) between pUC and vUC patients (46.6% vs. 46.2%, p = 0.97).

In our research, we found that the Bajorin score, along with the baseline WBC and hemoglobin levels, hold substantial significance as prognostic factors. These outcomes align with the observations made by Bajorin et al. during the pre-immunotherapy era, when cisplatin-based therapy was primarily the established treatment protocol [33]. Leukocytosis, as indicated in previous reports, may be linked to tumors that produce granulocyte colony-stimulating factor (G-CSF), and this association is correlated with a less favorable prognosis [34]. Similarly, anemia has been identified as an independent factor associated with reduced OS and CSS among urothelial carcinoma patients [35]. Although cancer-related anemia typically has multiple contributing factors, the interaction between leukocytosis and anemia can, in part, be elucidated by G-CSF’s impact on inhibiting bone marrow erythropoiesis and promoting splenic erythropoiesis, ultimately exacerbating anemia [36].

There are a few limitations that are inherent to the nature of the retrospective design of this study including lack of randomized comparisons, lack of external validation, heterogeneity of clinical practice and missing PD-L1 status for further investigation. Notably, a greater proportion of pUC patients received anti-PD1 therapy as compared to vUC with statistical significance, which may affect further survival outcome. Second, the assignment of patients to different treatment groups relied on the physician’s discretion and patient preferences, leading to inherent selection bias. However, the analysis revealed no discernible differences in demographic variables, including sex, age and Bajorin prognostic factors, suggesting that that the imbalance treatment bias was partially mitigated. Third, in clinical practice, a significant number of diagnoses of mUC rely on small core biopsy specimens. Nonetheless, the limited number of tumor cells present in these small biopsies, coupled with the intrinsic heterogeneity of tumors, can result in an underestimation of the true proportion of vUC cases. Fourth, up to 40% cases lack the PD-L1 values in this study. This was primarily due to the earlier commencement of our study (Apr 2016) before the Food and Drug Administration (FDA) announced restrictions on the front-line use of ICI for cisplatin-ineligible patients in June 2018. The balanced distribution of missing PD-L1 values helped mitigate the bias to some extent.

## Conclusion

In vUC patients, ICI monotherapy is an effective treatment option either as first line or chemotherapy resistant second-line treatment in advanced/metastatic UC. Future randomized prospective trial and response analysis of different histologic subtype to ICI is needed.

### Electronic supplementary material

Below is the link to the electronic supplementary material.

Supplementary Material 1

## Data Availability

The datasets used and/or analysed during the current study available from the corresponding author on reasonable request.

## Abbreviations

pUC: Pure urothelial carcinoma
vUC: Variant urothelial carcinoma
mUC: Metastatic urothelial carcinoma
ICI: Immune checkpoint inhibitor
TMB: Tumor mutation burden
